# Marine soundscape shaped by fishing activity

**DOI:** 10.1098/rsos.160606

**Published:** 2017-01-11

**Authors:** Laura Coquereau, Julie Lossent, Jacques Grall, Laurent Chauvaud

**Affiliations:** 1 Laboratoire des Sciences de l'Environnement Marin, Université de Bretagne Occidentale, Institut Universitaire Européen de la Mer, UMR 6539, LIA BeBEST, Rue Dumont D'Urville, 29280 Plouzané, France; 2 France Energies Marines, 15 Rue Johannes Kepler, Site du Vernis, Technopole Brest Iroise, 29200 Brest, France; 3 Observatoire Marin, UMS 3113, Institut Universitaire Européen de la Mer, Rue Dumont D'Urville, 29280 Plouzané, France

**Keywords:** soundscape, fishing, maerl beds, acoustic ecology, snapping shrimps

## Abstract

Marine communities face anthropogenic pressures that degrade ecosystems. Because underwater soundscapes carry information about habitat quality, we explored whether destructive impacts of fishing could be evaluated via the soundscape. Maerl beds are recognized as biodiversity hotspots and they experience major worldwide degradation owing to fishing. We collected field acoustic recordings in maerl beds exposed to different fishing practices. We found that unfished maerl beds were threefold louder and exhibited sound frequencies more diversified than those recorded in fished maerl beds. Analyses of associated fauna samples indicated that snapping shrimps provided a major contribution to the maerl bed soundscape. Moreover, sea urchins and squat lobsters most likely contributed to differences between the soundscapes of unfished and fished maerl beds. Our results supported the idea that the soundscape can provide valuable information on maerl bed ecosystem health related to fishing activity.

## 1. Introduction

Humans are currently altering marine and estuarine ecosystems at an unprecedented rate [1]. The development of non-destructive ecological assessment tools has become a priority for both biodiversity conservation and ecosystem management. The soundscape, investigated with non-intrusive passive acoustic methods is now recognized as a fundamental feature in ecology [2,3]. Ocean soundscapes provide important dynamic and sensory information about marine organisms in space and time, including their spatial orientations, habitat selections, reproduction and trophic interactions [2,4,5]. Previous studies showed that sound production could serve as proxies of marine health status [3,6,7]. Demersal fishing affects benthic communities [8] and could affect the soundscape, either directly, by influencing the sound-producing species assemblages, or indirectly, through changes in habitat structure. It remains to be assessed whether destructive fishing impacts can be evaluated based on changes in the soundscape.

Maerl beds comprise a highly vulnerable habitat that has experienced severe, worldwide degradation owing to fishing practices (trawling and dredging). Scientists and international authorities have turned their attention to this ‘hotspot’ of biodiversity, because of its ecological, economical and cultural interests [9,10]. Fishing practices can degrade maerl beds by reducing the maerl layer depth, disrupting bed surfaces, or even completely destroying the habitat [11]. These impacts lead to reduced habitat complexity and associated biodiversity. In this study, we assessed whether maerl bed soundscapes could reflect the impact of fishing.

## 2. Material and methods

Over 20 years (1992–2012), benthic invertebrates have been sampled from maerl habitats in the Bay of Brest, France. Among these maerl beds, we chose two that were similar in environmental properties and benthic community structures before 2004. After this year, intensive fishing practices began in one of these beds (the ‘fished bed’), but not the other (the ‘unfished bed’). The two beds are situated 1.8 km apart. We analysed a temporal series of data on macrofaunal species richness (number of species per sample) to characterize changes in maerl communities induced by high fishing pressure. Data consisted of three replicates of 0.1 m^2^ sediment samples collected in autumn using a 0.1 m^2^ Smith–McIntyre grab. After sorting, all animals were identified to the species level and numbered. In a context of study at the Institut Universitaire Européen de la Mer, no specific permission was required for the sampling or the recording in this study.

We conducted acoustic recordings in autumn 2014 (season with the maximum of biomass and abundance [12]) and spring 2015 (season following fishing activity in the winter) in these two maerl beds. We acquired recordings with a wide-band omnidirectional hydrophone (HTI-92-WB, High Tech Inc., sensitivity: −155 dB re 1 V µPa^−1^) connected to an EA-SDA14 autonomous recorder (RTSys®, sampling rate: 156′250 Hz, resolution: 24 bit, acquisition chain fully calibrated). The recording device was placed on the seafloor, supported by a weighted (15 kg) aluminium tripod, at a mean (±s.d.) water depth of 5.7 ± 1.5 m. The distance between the hydrophone and the seafloor was fixed at 1 m. We acquired two 10 min recordings (‘snapshot’ sound recordings as suggested in [6]), one during the daytime (from 10.00 to 16.00 h) and the other at dusk/early night, to capture potential diurnal variability in sound production [13]. Recordings were acquired at five sampling sites within each maerl bed (i.e. total of 10 recordings). These 10 recordings were conducted on the same day. Three sampling days (considered here as pseudo replicates) were conducted in each season to capture the natural seasonal variability of the benthic community structure and soundscape.

The audio files were analysed with Raven Pro 1.5®, and we applied specific signal processing routines developed in Matlab®. The replicates showed low variability in space (between the five sites in each bed) and time (between the three replicates within a given season); thus, we compiled recordings to generate mean measurements for the fished and the unfished maerl beds. The soundscapes were composed of sounds from two sets of sources: (i) multiple distant, indistinguishable sound sources, and (ii) a few high-energy, nearby, identifiable sound sources. The sound differences between the two beds were evaluated by measuring ambient noise levels (ANLs, in dB re µPa), which comprised the first set of sources. Recordings were divided into 10 s bins for which ANL was calculated according to the method described in [14]. Then these 10 s bins belonging to a 10 min recording were compiled to obtain an ANL value. Regardless of this analysis, we detected high-energy benthic pulses (the 95th percentile). For each one of them, we computed the peak frequency (*f*_p_, in Hz) and the corresponding sound pressure level (SPL, in dB re 1 µPa). Then, we plotted *f*_p_ as a function of SPL. The ANLs data were not normally distributed (Shapiro–Wilk's test *p* < 0.05); therefore, we used a non-parametric Wilcoxon's signed-rank test to evaluate differences between the two maerl beds for each season.

For a better ecological understanding of the acoustic variability between the fished and the unfished maerl beds, we examined quantitative fauna samples that targeted soniferous species. Scuba divers collected samples from one site per maerl bed, the day after each acoustic recording, resulting in three replicates per bed and per season. The species sampling list was based on a previous study that investigated sound-producing invertebrates living in the maerl beds of this region [15]. Additional details on fauna sampling are provided in the electronic supplementary material, S1. Maerl thickness and cover were estimated based on three replicates at each acoustic recording site with, respectively, for thickness and cover, 5.4 cm diameter cores and pictures with a 50 cm^2^-quadrat placed on the sediment, photographed and then further visually estimated in the laboratory.

## 3. Results

**Figure 1.**
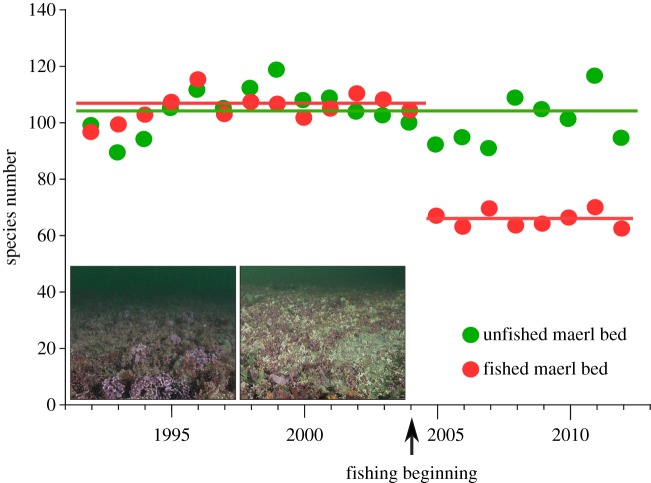
Invertebrate species richness in two maerl beds exposed to different fishing practices: no fishing (green dots); fishing (red dots), beginning in 2004 (arrow). Each point represents the mean number of species identified in three replicate sediment samples (each 0.1 m^−2^) collected in autumn with a Smith–McIntyre grab. The lines show the average of all points over the indicated time. Insets show images of the maerl bed surface, acquired from unfished (left) and fished (right) regions.

Analyses of macrofaunal samples collected over the past 20 years showed uniform species richness in both unfished maerl beds until 2004. After this year, the fished maerl bed showed an abrupt drop in mean species richness from 105 to 66 (figure 1). Time series dataset of species richness is provided in the electronic supplementary material, S2. The soniferous community samples were markedly different between the two maerl beds (table 1). The abundance and richness of acoustic species were higher in the unfished bed (49 ± 17 m^−2^ and 6 ± 3 m^−2^, respectively) than in the fished bed (8 ± 5 m^−2^ and 3 ± 1 m^−2^, respectively).

**Table 1.** Differences in soniferous benthic invertebrate and seabed characteristics between an unfished- and a fished maerl bed in Brittany, France.

	unfished bed	fished bed
soniferous species abundances (n m^−2^)		
*Athanas nitescens*	48	8
*Galathea squamifera*	8	—
*Maja brachydactyla*	1	1
*Paracentrotus lividus*	2	—
*Pecten maximus*	1	—
*Psammechinus miliaris*	4	2
mean soniferous species abundances (n m^−2^)	64	11
mean soniferous species richness/sample	6	3
maerl cover (%)	100	55
maerl thickness (mm)	42	4

**Figure 2.**
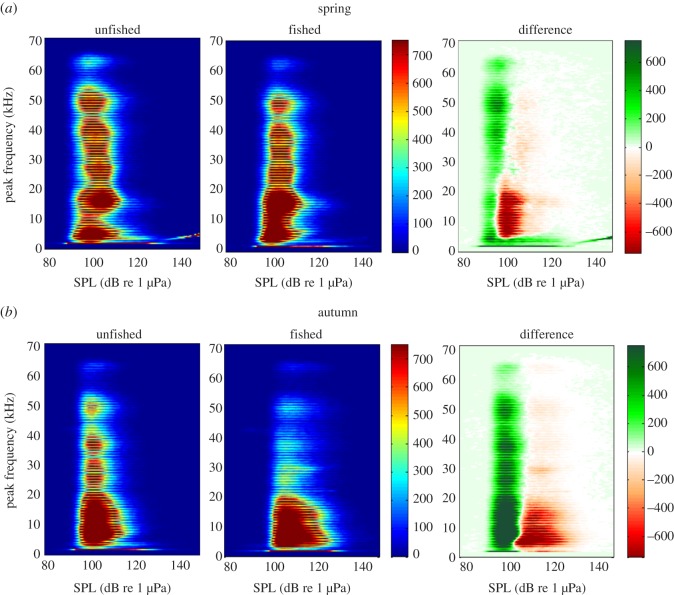
Peak frequencies (*f*_p_) at different sound pressure levels (SPL) in two maerl beds exposed to different fishing practices. Data represent the 95th percentile of benthic pulses (*n* > 3 × 10^5^ per graph), recorded during spring (*a*) and autumn (*b*). Colour scales indicate benthic pulse number at a given *f*_p_ and SPL. Differences were calculated as: unfished benthic pulse number − fished benthic pulse number. Colour bars correspond to benthic pulse number.

In both seasons, the mean ANLs were significantly higher in the unfished bed (spring: 91.8 ± 1.6 dB re 1 µPa; autumn: 98.1 ± 2.5 dB re 1 µPa) than in the fished bed (spring: 83.6 ± 3.2 dB re 1 µPa, *p* = 0.022; autumn: 91.3 ± 3.0 dB re 1 µPa, *p* = 0.007). ANL spectra of the two maerl beds and spectrograms are provided in the electronic supplementary material, S3 and S4, respectively. The plots of *f*_p_ as a function of SPL showed a more diversified *f*_p_ in the unfished bed than in the fished bed, with a stronger effect in autumn (figure 2). Compared with the fished bed, the unfished bed exhibited more pulses at *f*_p_s of 27, 37, 49 and 63 kHz. We also observed the presence of higher SPLs in the fished bed than in the unfished bed, particularly at *f*_p_s of 3–15 kHz.

## 4. Discussion

Our results showed that dredging strongly altered the marine soundscape. It has been claimed that demersal gears (dredges and trawls) affect marine habitats like clear-cutting affects forests, in terms of the damage incurred to biodiversity and habitat structure [11]. By conducting field recordings, we found that the mean ANL in a fished maerl bed was threefold lower than that in an unfished bed. Furthermore, the diversity of sound frequencies appeared to be impoverished in the fished bed, with gaps at several frequency levels. These results demonstrated the importance of including high frequencies in the bandwidth for distinguishing altered soundscapes in beds disturbed by fishing practices; an analysis at low frequency bandwidths would be less discriminating. The presence of high SPL values in the fished bed compared with the unfished bed might be explained by the greater thickness of maerl in the unfished bed. Thick beds might attenuate sounds from animals that live within the maerl matrix; by contrast, more animals lived above the thin matrix in the fished bed [16]. Overall, the changes we observed in acoustic features, i.e. higher-quality habitats are louder and richer in acoustic events than degraded ones, were consistent with observations in other marine habitats [3,6]. Nevertheless, these comparative studies of habitat quality through soundscape are prone to the confounding effects of variables such as habitat type, anthropogenic impact and environmental parameters (e.g. tides). The advantage of this study was that fishing was the only significantly different variable between the maerl beds; thus, the soundscape changes we observed were solely because of fishing practices.

Our data on associated benthic communities improve our understanding of soundscape variability between unfished and fished maerl beds. We found a greater diversity of sound sources in the unfished bed than in the fished bed. This finding suggested that, when more acoustic species lived in a maerl bed, the soundscape became more complex. This notion supported the ‘niche hypothesis’ of Krause [17], which held that a healthy ecosystem soundscape would show a uniform distribution of frequency niches, and that a disturbed ecosystem soundscape would show gaps at frequencies where species had been lost. Our fauna results clearly showed a high abundance of snapping shrimps *Athanas nitescens*, which produce sounds in the *f*_p_ range of 5–11 kHz [15], *f*_p_ found in both unfished and fished beds. Other acoustic sources contributed to the differences in frequency diversity between the two soundscapes. The sea urchins, *Psammechinus miliaris* and *Paracentrotus lividus*, could participate to these differences because they produce sounds in the *f*_p_ range of 39–49 kHz [15], and they were, respectively, eight and threefold more abundant, in the unfished than in the fished maerl beds. Another species that could be responsible of these differences in frequency diversity is the squat lobster, *Galathea squamifera*, present only in unfished bed samples and producing sounds at an *f*_p_ of about 25 kHz [18].

Measuring ecosystem health relies mainly on species inventories, which demand considerable sampling effort and mostly involve invasive methods. We showed that a non-invasive study of biological sound production provided valuable information on habitat degradation owing fishing. Moreover, we found that maerl bed soundscape patterns were maintained throughout spring and autumn, even if differences between unfished and fished beds seem more pronounced in autumn. Thus, soundscapes represent a stable, efficient, reliable indicator of habitat changes, for evaluating the fishing footprint.

Although our analysis was limited to northeastern Atlantic maerl beds, our strategy on soundscape and soniferous diversity modifications owing to fishing could be applicable to biogenic coastal assemblages found in any dredged/trawled habitats. Our findings also raised intriguing questions about implications on larval orientation in such habitats that act as a nursery for fishes and invertebrates [9], because the underwater soundscape plays a critical role in larval settlement [19,20]. How these modifications might affect larval orientation in maerl beds remains to be explored.

## Supplementary Material

ESM 1 - Fauna sampling. Fauna sampling associated with acoustic recordings

## Supplementary Material

ESM 2 - Time series data set of species richness. Time series data set of the invertebrate species richness over 20 years in maerl beds exposed to different fishing practices

## Supplementary Material

ESM 3 - ANL spectra. ANL spectra of the unfished (green line) and fished (red line) maerl beds recorded in the Bay of Brest. Grey lines indicate Wenz's wind and traffic noise curves (Wenz 1962)

## Supplementary Material

ESM 4 - Spectrograms. Spectrograms of the unfished (left) and fished (right) maerl beds recorded in spring in the Bay of Brest

## Data accessibility

All relevant data are within the paper and the electronic supplementary material.
