# Correction to ‘Marine soundscape shaped by fishing activity’

**DOI:** 10.1098/rsos.170554

**Published:** 2017-06-21

**Authors:** Laura Coquereau, Julie Lossent, Jacques Grall, Laurent Chauvaud

*R. Soc. open sci*. **4**, 160606. (Published 11 January 2017). (doi:10.1098/rsos.160606)

This correction is to expand upon on the electronic supplementary material presented in the manuscript.

In the corresponding electronic supplementary material file, additional information about acoustic calculations and data collection has been provided (https://dx.doi.org/10.6084/m9.figshare.c.3653213).

Additionally, 10-min sample recordings of both sample sites have been made available via Dryad: http://dx.doi.org/10.5061/dryad.s6f4q.

## Supplementary Material

Supplementary File 5

