# Supplementary material for: Marine soundscape shaped by fishing activity
Source: R Soc Open Sci. 2017 Jan 11;4(1):160606. doi: 10.1098/rsos.160606 (PMC5319325; doi:10.1098/rsos.160606)
Supplement: ESM 1 - Fauna sampling. Fauna sampling associated with acoustic recordings [file rsos160606supp1.pdf]

Marine soundscape shaped by fishing activity

Laura Coquereau [1,*], Julie Lossent [2], Jacques Grall [3], Laurent Chauvaud [1,3]

[1]*Université de Bretagne Occidentale, Institut Universitaire Européen de la Mer, Laboratoire des Sciences de l'Environnement Marin, UMR 6539, LIA BeBEST, Rue Dumont D'Urville, 29280 Plouzané, France*

[2]*France Energies Marines, 15 rue Johannes Kepler, Site du Vernis, Technopole Brest Iroise, 29200 Brest, France*

[3]*Observatoire Marin, UMS 3113, Institut Universitaire Européen de la Mer, Rue Dumont D'Urville, 29280 Plouzané, France*

* Corresponding author

E-mail address: laura.coquereau@univ-brest.fr

Fauna sampling associated with acoustic recordings

To better understand acoustic variability in the fished and the unfished maerl beds, quantitative soniferous fauna samples were collected. The species sampling list was based on a previous study investigating sound-producing invertebrates living in maerl beds of this region (Coquereau et al, 2016). Highly mobile soniferous megafauna, i.e. *Maja brachydactyla*, *Echinus esculentus* and *Pecten maximus,* was collected on a surface sampling of 80 m², whereas less mobile soniferous megafauna species, i.e. *Paracentrotus lividus* and *Psammechinus miliaris,* was sampled on a surface of 20 m². The surfaces were measured by a rope that divers used to make the radius of a circle (5 m and 2.5 m). The fauna was release at sea after identification and counting. To sample the small soniferous species *Athanas nitescens*, *Crepidula fornicata* and *Mimachlamys varia,* a fauna extraction by suction with a device equipped with a 0.25 m² quadrat and a net of 1 mm mesh size was used by the divers. Five suction replicates were taken at the same site as the megafauna sampling in each maerl bed. Abundances were converted to units per m².
