# Supplementary material for: Marine soundscape shaped by fishing activity
Source: R Soc Open Sci. 2017 Jan 11;4(1):160606. doi: 10.1098/rsos.160606 (PMC5319325; doi:10.1098/rsos.160606)
Supplement: ESM 2 - Time series data set of species richness. Time series data set of the invertebrate species richness over 20 years in maerl beds exposed to different fishing practices [file rsos160606supp2.pdf]

Marine soundscape shaped by fishing activity

Laura Coquereau [1,*], Julie Lossent [2], Jacques Grall [3], Laurent Chauvaud [1,3]

[1]*Université de Bretagne Occidentale, Institut Universitaire Européen de la Mer, Laboratoire des Sciences de l'Environnement Marin, UMR 6539, LIA BeBEST, Rue Dumont D'Urville, 29280 Plouzané, France*

[2]*France Energies Marines, 15 rue Johannes Kepler, Site du Vernis, Technopole Brest Iroise, 29200 Brest, France*

[3]*Observatoire Marin, UMS 3113, Institut Universitaire Européen de la Mer, Rue Dumont D'Urville, 29280 Plouzané, France*

* Corresponding author

E-mail address: laura.coquereau@univ-brest.fr

Time series data set of the invertebrate species richness over 20 years in maerl beds exposed to different fishing practices

Year	Fished bed	Unifished bed
1992	97.0	99.3
1993	100.0	89.7
1994	103.0	94.7
1995	105.0	105.0
1996	115.0	111.3
1997	103.0	104.7
1998	109.0	111.3
1999	107.0	120.0
2000	101.3	109.3
2001	105.0	109.0
2002	110.7	104.0
2003	108.3	103.3
2004	103.7	100.0
2005	66.3	92.7
2006	63.0	94.3
2007	69.7	91.3
2008	63.0	108.7
2009	64.7	105.3
2010	66.7	101.0
2011	70.0	118.0
2012	62.7	94.7
