# Supplementary material for: Marine soundscape shaped by fishing activity
Source: R Soc Open Sci. 2017 Jan 11;4(1):160606. doi: 10.1098/rsos.160606 (PMC5319325; doi:10.1098/rsos.160606)
Supplement: ESM 3 - ANL spectra. ANL spectra of the unfished (green line) and fished (red line) maerl beds recorded in the Bay of Brest. Grey lines indicate Wenz's wind and traffic noise curves (Wenz 1962) [file rsos160606supp3.pdf]

Marine soundscape shaped by fishing activity

Laura Coquereau [1,*], Julie Lossent [2], Jacques Grall [3], Laurent Chauvaud [1,3]

[1]*Université de Bretagne Occidentale, Institut Universitaire Européen de la Mer, Laboratoire des Sciences de l'Environnement Marin, UMR 6539, LIA BeBEST, Rue Dumont D'Urville, 29280 Plouzané, France*

[2]*France Energies Marines, 15 rue Johannes Kepler, Site du Vernis, Technopole Brest Iroise, 29200 Brest, France*

[3]*Observatoire Marin, UMS 3113, Institut Universitaire Européen de la Mer, Rue Dumont D'Urville, 29280 Plouzané, France*

* Corresponding author

E-mail address: laura.coquereau@univ-brest.fr

ANL spectra of the unfished (green line) and fished (red line) maerl beds recorded in the Bay of Brest. Grey lines indicate Wenz's wind and traffic noise curves (Wenz 1962).

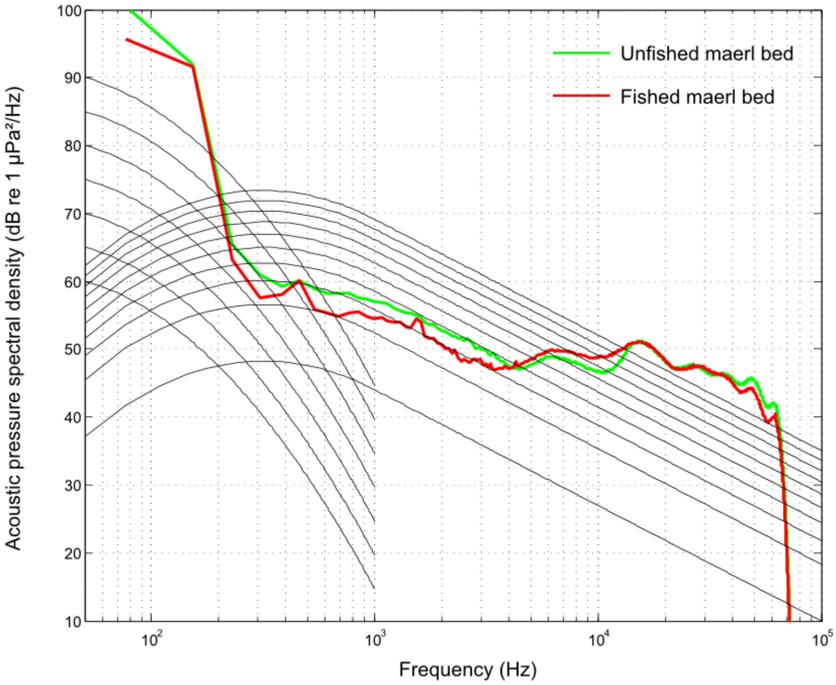

Wenz (1962) Acoustic ambient noise in the ocean: spectra and sources. J Acoust Soc Am 34:1936-1956
