# Supplementary material for: Marine soundscape shaped by fishing activity
Source: R Soc Open Sci. 2017 Jan 11;4(1):160606. doi: 10.1098/rsos.160606 (PMC5319325; doi:10.1098/rsos.160606)

# Marine soundscape shaped by fishing activity

Laura Coquereau [1,*], Julie Lossent [2], Jacques Grall [3], Laurent Chauvaud [1,3]

[1]*Université de Bretagne Occidentale, Institut Universitaire Européen de la Mer, Laboratoire des Sciences de l'Environnement Marin, UMR 6539, LIA BeBEST, Rue Dumont D'Urville, 29280 Plouzané, France*

[2]*France Energies Marines, 15 rue Johannes Kepler, Site du Vernis, Technopole Brest Iroise, 29200 Brest, France*

[3]*Observatoire Marin, UMS 3113, Institut Universitaire Européen de la Mer, Rue Dumont D'Urville, 29280 Plouzané, France*

* Corresponding author

E-mail address: laura.coquereau@univ-brest.fr

Spectrograms of the unfished (left) and fished (right) maerl beds recorded in spring in the Bay of Brest.

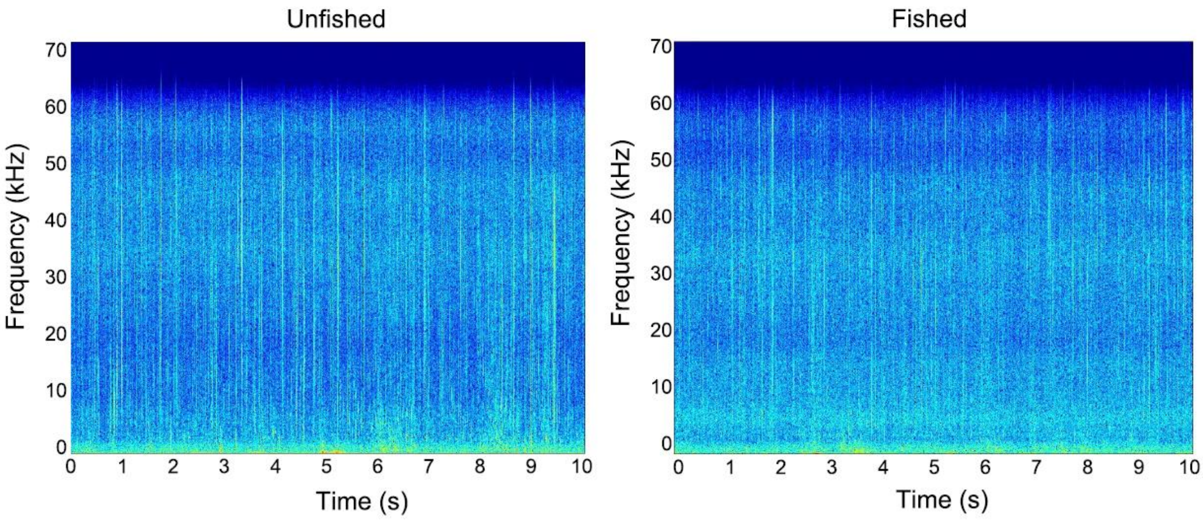

Supplement: ESM 4 - Spectrograms. Spectrograms of the unfished (left) and fished (right) maerl beds recorded in spring in the Bay of Brest [file rsos160606supp4.pdf]
