# Supplementary material for: Correction to ‘Marine soundscape shaped by fishing activity’
Source: R Soc Open Sci. 2017 Jun 21;4(6):170554. doi: 10.1098/rsos.170554 (PMC5493934; doi:10.1098/rsos.170554)
Supplement: Supplementary File 5 [file rsos170554supp1.pdf]

Marine soundscape shaped by fishing activity

Laura Coquereau [1,*], Julie Lossent [2], Jacques Grall [3], Laurent Chauvaud [1,3]

[1]*Université de Bretagne Occidentale, Institut Universitaire Européen de la Mer, Laboratoire des Sciences de l'Environnement Marin, UMR 6539, LIA BeBEST, Rue Dumont D'Urville, 29280 Plouzané, France*

[2]*France Energies Marines, 15 rue Johannes Kepler, Site du Vernis, Technopole Brest Iroise, 29200 Brest, France*

[3]*Observatoire Marin, UMS 3113, Institut Universitaire Européen de la Mer, Rue Dumont D'Urville, 29280 Plouzané, France*

* Corresponding author

E-mail address: laura.coquereau@univ-brest.fr

Additional acoustic information

DATA COLLECTION:

The same recording device has been used in the study Coquereau, L., Grall, J., Chauvaud, L., Gervaise, C., Clavier, J., Jolivet, A., & Di Iorio, L. (2016). Sound production and associated behaviours of benthic invertebrates from a coastal habitat in the north-east Atlantic. Marine Biology, 163(5), 1-13. The following figure (published in the previous reference) shows the recording device. It was deployed at predefined positions from a vessel. During recordings, the boat engine was shut off and the boat left to drift.

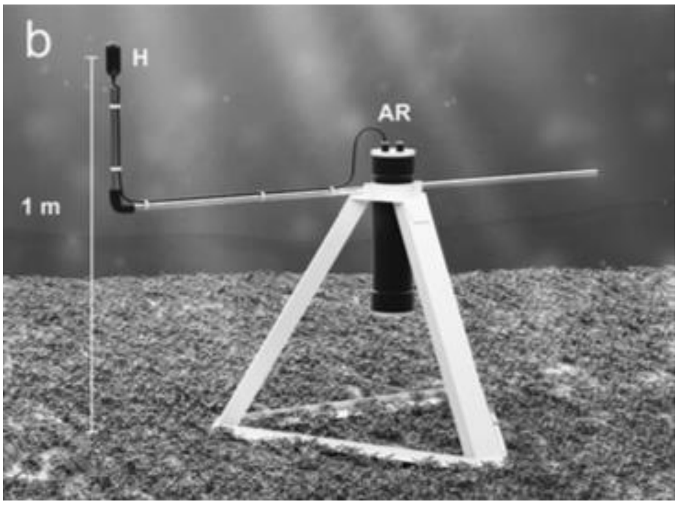

COMPUTATION

The audio files were analysed with homemade specific signal processing routines developed in Matlab®. Recordings were divided into 10-second bins segments. For each segment, a spectrogram was calculated (LFFT = 2048, Hanning window, overlap 50%). It contains 1524

spectra. The spectrograms were visually inspected and the segments containing boat noise were removed.

For each 10s segment, the Ambient Noise Level (ANL) was calculated according to the method described by Kinda et al. (2013) in the Appendix (Kinda GB, Simard Y, Gervaise C, Mars J, Fortier L. 2013 Under-ice ambient noise in Eastern Beaufort Sea, Canadian Arctic, and its relation to environmental forcing. J. Acoust. Soc. Am. 134, 77–87). We choose a percentile value of p = 0.2. After sorting each frequency line of the spectrogram of 10s, we estimated for each 10s segment the values at the chosen percentile. The ANL value of a 10s segment corresponds to the mean of the values obtained for each frequency bin within this 10s segment. Then, to obtain an ANL value for each position of measurement (10-minute recording on each site), we calculated a mean value from all the 10-s bins' ANL belonging to a 10 min recording.

Regardless of this ANL analysis, we detected high-energy benthic pulses (the 95th percentile) and estimated two features to characterize them.
Assuming the hypothesis that the signal's envelop follows a Gaussian law, our algorithm detected the rising fronts of the signals with a signal to noise ratio of 7 dB and associated each rising front to a pulse's time of arrival at the hydrophone. The Sound Pressure Level (SPL, dB re. 1µPa) and peak frequency (f_p, in Hz) were calculated on the first maxima after the rising front of each pulse. We used for the analysis only the benthic pulses, whose SPL was within the loudest 5% (95th percentile).
